# Why it’s better to believe in a larger definition of the diametaphyseal junction zone in pediatric distal radius fractures

**DOI:** 10.1007/s00402-026-06428-8

**Published:** 2026-07-20

**Authors:** Christoph von Schrottenberg, Susann Marie Beck, Philipp Schwerk, Guido Fitze, Jurek Schultz

**Affiliations:** 1 Department of Pediatric Surgery, Faculty of Medicine and University Hospital Carl Gustav Carus, TUD Dresden University of Technology, Dresden, Germany; 2 Department of Pediatric Surgery and Pediatric Urology, City Hospital Chemnitz, Chemnitz, Germany

**Keywords:** Diametaphyseal radius fracture, Pediatric forearm fracture, Distal radius fracture, Children

## Abstract

**Introduction:**

Diametaphyseal radius fractures (DMRF) in children are characterized by special biomechanical behavior. They present with a stereotypical loss of reduction (radial translation and ulnar tilt) of the distal fragment when stabilized with elastic stable intramedullary nailing (ESIN), the standard osteosynthesis for pediatric diaphyseal forearm fractures. No consensus has been found on the definition of the diametaphyseal junction zone (DMJZ). Some authors claim that diaphyseal fractures immediately proximal to the metaphysis, as defined by the AO Pediatric Comprehensive Classification of Long Bone Fractures (AO-PCCF), can safely be stabilized with ESIN. They confine the DMJZ to a small part within the metaphysis. Other definitions of the DMJZ extend slightly more proximally, including a small part of the distal diaphysis. The forearm fracture index (FFI) calculates the ratio of the fracture’s distance to the radius’ growth plate over its width and defines DMRF to have an FFI between 1 and 2. The aim of this case series was to demonstrate that these particular fractures, which can be considered diaphyseal according to the AO-PCCF, still exhibit biomechanical characteristics typical of DMRF.

**Materials and methods:**

Radiologic and clinical outcomes of 11 DMRF, that would be considered diaphyseal by the AO-PCCF classification but fall into the DMJZ when using the FFI, are being reported.

**Results:**

7 fractures were treated with ESIN and 4 with transepiphyseal percutaneous intramedullary Kirschner-wire (TEPIK) fixation. ESIN osteosynthesis led to only a small reduction in angulation but a notable increase in translational dislocation in the a.p. radiograph, both typical dislocation patterns seen in DMRF. Fractures stabilized with TEPIK, an osteosynthesis technique obeying the biomechanical demands of DMRF, did not present with these stereotypical dislocation patterns.

**Conclusions:**

The biomechanical demands typical of DMRF also apply to very distal diaphyseal forearm fractures. Hence, a larger definition of the DMJZ might include all fractures with the characteristic biomechanical behavior.

**Supplementary Information:**

The online version contains supplementary material available at 10.1007/s00402-026-06428-8.

## Introduction

**Fig. 1:**
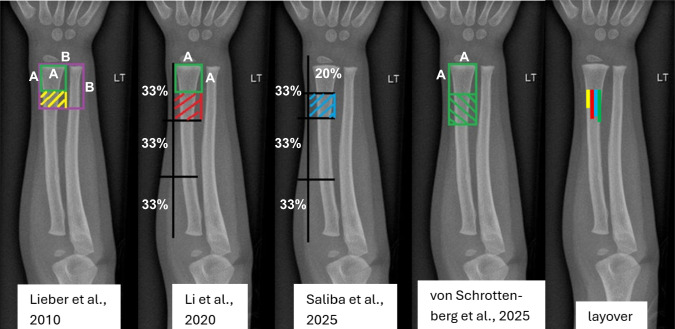
**a**–**e** Visualization of 4 different definitions of the diametaphyseal junction zone. **a** the yellow area defines the DMJZ according to Lieber et al. [2]; **b** the red area defines the DMJZ according to Li et al. [9]; the black lines divide the radius into 3 thirds, without the epiphyses. **c** the blue area defines the DMJZ according to Saliba et al. [7]; here, the black line also divides the radius into 3 thirds, including the epiphyses. **d** the green area defines the DMJZ according to von Schrottenberg et al. [13]; **e** the length of the 4 different definitions of the DMJZ are applied, visualizing length discrepancies

Diametaphyseal radius fractures (DMRF) in children are subject to ongoing investigations [1]. Numerous surgical techniques that meet the specific biomechanical challenges of these fractures have been published [2–7]. Still, definitions of the diametaphyseal junction zone (DMJZ) are heterogeneous amongst studies [2, 7–9]. The most commonly used definition places the DMJZ completely inside the metaphysis, as defined by the AO Pediatric Comprehensive Classification of Long Bone Fractures (PCCF) [2, 10–12]. No part of the diaphysis is included in this DMJZ. In 2025, an investigational approach to define the DMJZ using the forearm fracture index (FFI) was introduced. The FFI is defined as the ratio of the fracture’s distance to the distal radius’ growth plate over its width [13]. Fractures with an FFI between 1 and 2 are considered DMRF. This definition extends the DMJZ more proximally and includes a small part of the distal diaphysis according to the AO-definition, leading to an increase of DMRF by 6% when compared to the most commonly used definition by Lieber et al. [13]. Definitions of the DMJZ published by Saliba et al. and Li et al. lie between these 2 definitions [7, 9]. Figure 1 visualizes 4 definitions of the DMJZ and compares the length of the included part of the radius. Table 1 summarizes the characteristics of these 4 definitions and 1 other published by Kim et al. [8].

**Table 1:** Overview of 5 different definitions of the diametaphyseal junction zone (DMJZ) including advantages and disadvantages

Author	Description	Requirements	Advantages	Disadvantages
Lieber et al. [2]	The part of the metaphysis proximal to the square over the radius growth plate alone	a.p. radiograph of the wrist	If applied in the lateral radiograph, the fracture's true distance to the growth plate can be assessed	May underestimate the fracture's true distance to the growth plate if only the a.p. radiograph is assessed
			Easy to calculate	No exact localization of the fracture within the DMJZ
			Widely used	Some fractures proximal to the DMJZ still demonstrate biomechanical behavior typical for DMRF
Kim et al. [8]	“Fracture[s] with (1) the distance between the fracture line and the distal articular surface between 35 and 60 mm; (2) the ratio of the length of distal fragment to the total length of radius within 25%; and (3) the ratio of the maximal diameter at 2 cm proximal to the fracture line to that at 2 cm distal to the fracture line within 70%”	a.p. radiograph of the entire forearm	Independent of growth plates	Radiograph of the entire forearm is needed
				No exact localization of the fracture within the DMJZ
				Complicated to calculate
Li et al. [9]	The distal third of the radius minus the square of the width of the radius growth plate	a.p. radiograph of the entire forearm		Radiograph of the entire forearm is needed
				No exact localization of the fracture within the DMJZ
Saliba et al. [7]	The distal third of the radius minus the distal fifth	a.p. radiograph of the entire forearm	Independent of growth plates	Radiograph of the entire forearm is needed
				No exact localization of the fracture within the DMJZ
von Schrottenberg et al. [13]	Fractures with an FFI between 1 and 2 are considered DMRF. The FFI is calculated by the fracture's true distance to the growth plate (assessed in the lateral radiograph) divided by the width of the radius growth plate (assessed in the a.p. radiograph)	a.p. and lateral radiograph of the wrist. If no lateral radiograph exists, the fracture's distance can be estimated from the a.p. radiograph	If applied in the lateral radiograph, the fracture's true distance to the growth plate can be assessed	Relatively unknown
			Exact localization of the fracture within the DMJZ	May underestimate the fracture's true distance to the growth plate if only the a.p. radiograph is assessed
			Enables objective comparison of studies/fractures	
			Applicable via ultrasound	
			Easy to calculate	

*DMRF* Diametaphyseal radius fracture, *a.p.* Anterior-posterior, *FFI* Forearm fracture index, Kim et al.’s definition is not included in Fig. 1

Although the FFI is straightforward to apply and offers the possibility of pinpointing the exact location of DMRF with an index that can make studies objectively comparable, it has not been widely adopted by many clinicians so far. Critics of this approach to defining the DMJZ claim that it includes too large a part of the radius. They argue that conventional ESIN osteosynthesis, applicable to diaphyseal forearm fractures, can be used in fractures located in the proximal part of this larger definition of the DMJZ without loss of reduction. This study aims to investigate whether fractures, categorized as diaphyseal according to the AO-PCCF, but considered to lie within the DMJZ as defined by an FFI between 1 and 2, can be stabilized with the conventional ESIN technique designed for diaphyseal shaft fractures [10–12, 14–16]. Radiographic reduction results after conventional ESIN osteosynthesis are analyzed and compared to results after transepiphyseal percutaneous intramedullary Kirschner-wire (TEPIK) fixation in these particular fractures.

## Methods

**Fig. 2:**
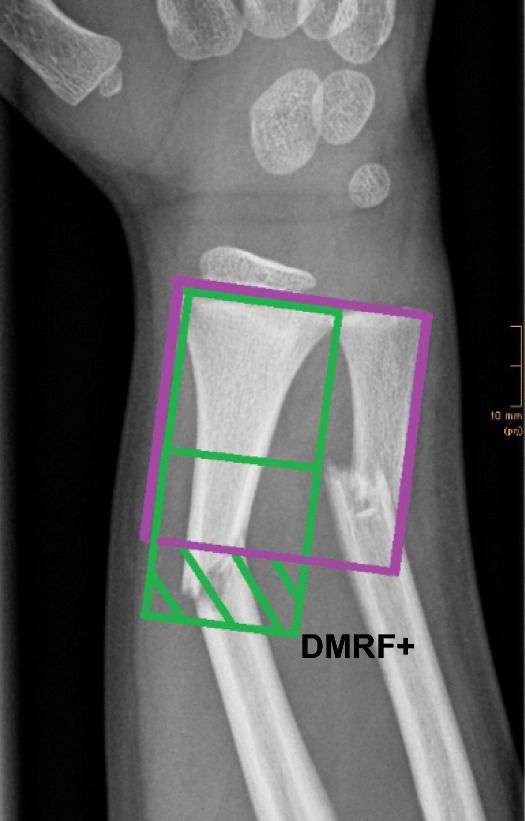
Visualization of the metaphysis according to the AO-definition (purple square); the proximal green square defines the diametaphyseal junction zone (DMJZ). Fractures in this area have a forearm fracture index between 1 and 2. The dashed area displays the area within this DMJZ proximal to the metaphysis. Fractures in this particular area can be named DMRF+

After approval from the local ethics committee (EK 433102016) was obtained and informed consent was waived due to the retrospective nature of the study, all forearm fractures in patients aged 16 years or younger treated at our institution between 2010 and 2020 were analyzed. ICD-Codes S52.0 – S52.9 were included. Duplicates, falsely coded, and pathological fractures, solitary fractures of the ulna, and patients with closed growth plates were excluded. Fractures with an FFI between 1 and 2 were identified as diametaphyseal and included. Buckle fractures, fractures lost to follow-up, and conservatively treated fractures were then excluded, as well as fractures that were stabilized with plate osteosynthesis or bicortical Kirschner-wire fixation. Fractures with an FFI between 1 and 2, that could be categorized as diaphyseal according to the AO-PCCF definition of the diaphysis, were defined as DMRF+ (see Fig. 2) and included; all other DMRF distally were excluded [10–13]. A flow diagram of patient acquisition is provided in the Supplementary Material (Fig. S1).

A retrospective analysis concerning radiographic and clinical outcomes of this case series, with exploratory comparison between patients treated with TEPIK and ESIN, was performed. Indication for surgical stabilization was usually given in severely displaced or unstable fractures. The choice of osteosynthesis lay at the discretion of the attending surgeon on call and was not subject to randomization. Initial dislocation and postoperative reduction results were assessed with the following 4 variables: angulation in the a.p. and lateral radiograph in degrees, translation in the a.p. and lateral radiograph as a percentage of the shaft’s width. Data was tested for normality using the Shapiro-Wilk test. A comparison of matched pairs was performed using the paired t-test when the data were normally distributed, and the results were displayed as mean values with standard deviations (SDs). Significance was set at .05. GraphPad Prism version 8.4.3 for Windows, GraphPad Software, San Diego, California, USA was used for data curation and statistical analysis.

## Results

### Patient acquisition and demographics

Of 516 DMRF identified with an FFI between 1 and 2, 132 buckle fractures and 18 fractures that were lost to follow-up were excluded. 270 were treated conservatively and hence excluded. 21 fractures that were stabilized with plate osteosynthesis (n = 5) and bicortical Kirschner-wire fixation (n = 16) were also excluded. Of the remaining 75 patients, 11 DMRF (6 male, 5 female) met the criteria of DMRF+ and were hence included in this analysis. 7 fractures had been stabilized with ESIN and 4 fractures with TEPIK. Patients’ mean age was 8.5 ± 3.8 years and the mean FFI was 1.77 ± 0.14. Median radiologic follow-up was 85 days (IQR, 69–95 days). In all patients but one, the clinical course was uneventful with good functional outcomes and no complications. One 14-year-old female patient stabilized with ESIN developed a pseudarthrosis and presented with persisting deficiency of the forearm rotation (supination/pronation: 40–0–90°) 1.5 years after the accident. Table 2 displays the cohort’s demographics and fracture characteristics.

**Table 2:** Demographic data and fracture characteristics of our cohort of 11 patients with DMRF+ stabilized with either the conventional elastic stable intramedullary nailing (ESIN) technique or with transepiphyseal percutaneous intramedullary Kirschner-wire fixation (TEPIK)

Sex	Age (years)	Greenstick	Concomitant ulna fracture	FFI	Radiologic follow-up (days)	Osteo-synthesis
Male	6	No	Yes	1.7	76	ESIN
Male	4	Yes	Yes	1.9	186	ESIN
Female	14	No	Yes	1.9	357	ESIN
Male	4	Yes	Yes	1.7	85	ESIN
Male	14	Yes	Yes	1.6	95	ESIN
Female	8	No	Yes	1.9	94	ESIN
Male	5	Yes	No	1.7	74	ESIN
Female	7	No	No	1.96	27	TEPIK
Female	9	No	Yes	1.89	31	TEPIK
Male	13	No	Yes	1.97	90	TEPIK
Female	10	No	Yes	1.95	69	TEPIK

*FFI* Forearm fracture index, DMRF+, fractures with an FFI between 1 and 2 but considered diaphyseal according to the AO-PCCF definition

### Analysis of dislocation patterns before and after ESIN osteosynthesis

Analysis of dislocation patterns of DMRF+ before and after ESIN osteosynthesis revealed the following: Angulation of the distal radius fragment in the a.p. radiograph was reduced from 17° (±12°) to 7° (±6°) (*p* = 0.1128). Angulation in the lateral radiograph was reduced from 37° (±17°) to 3° (±3°) (*p* = 0.0032). Radial translation of the distal radius fragment in the a.p. radiograph increased from 5% (±9%) to 35% (±27°) (*p* = 0.0284). No translation of the distal radius fragment to the ulnar side was observed. One patient presented with a 100% translation of the distal fragment in the lateral radiograph, which was fully reduced (0%).

### Analysis of dislocation patterns before and after TEPIK osteosynthesis

**Fig. 3:**
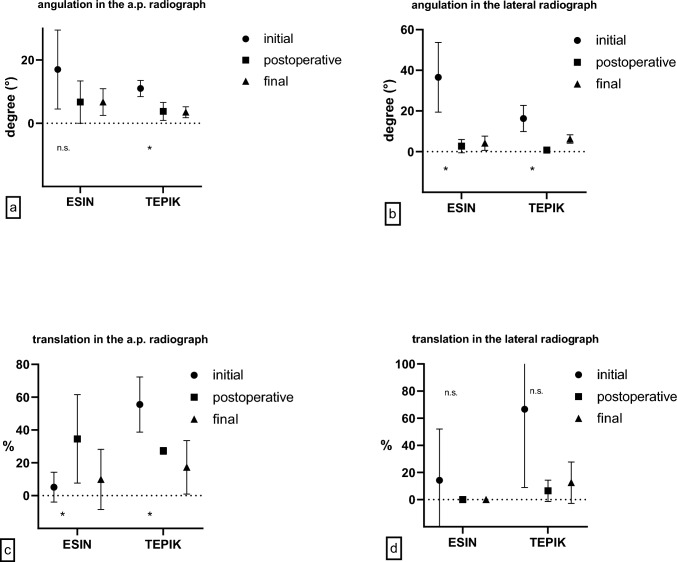
**a**–**d** Dislocation patterns of the distal radius fragment initially, after reduction and ESIN or TEPIK osteosynthesis and at the final radiologic follow-up **a** angulation in the a.p. radiograph; **b** angulation in the lateral radiograph; **c** translation as percentage of the shaft’s width in the a.p. radiograph; **d** translation as percentage of the shaft’s width in the lateral radiograph; Note how in **c** the percentage of translation from the initial radiograph to after reduction and ESIN osteosynthesis increases. This illustrates the biomechanical character of these fractures leading to a radial translation and an ulnar deviation of the distal radius fragment when the conventional ESIN technique is applied. n.s., not significant; *, *p* < 0.05

Analysis of dislocation patterns of DMRF+ before and after TEPIK osteosynthesis revealed the following: Angulation of the distal radius fragment in the a.p. radiograph was reduced from 11° (±2°) to 4° (±3°) (*p* = 0.0167). The direction of angulation was equally distributed towards the ulnar and radial side. Angulation in the lateral radiograph was reduced from 17° (±6°) to 1° (±1°) (*p* = 0.048). Translation of the distal radius fragment in the a.p. radiograph decreased from 56% (±17%) to 27% (±2%) (*p* = 0.0355). Median translation in the lateral radiograph was reduced from 67% (±58%) to 6% (±7%) (*p* = 0.1846). Figure 3 visualizes the various dislocation patterns throughout treatment, comparing ESIN with TEPIK.

These results illustrate that conventional ESIN osteosynthesis may increase translational displacement of the distal fragment in DMRF+ (see Fig. 3c). While angulation in the lateral radiograph was reduced, angulation in the a.p. radiograph was not reduced. Interestingly, 3 out of 7 fractures initially presented with ulnar deviation, but 5 out of 7 after osteosynthesis.

**Fig. 4:**
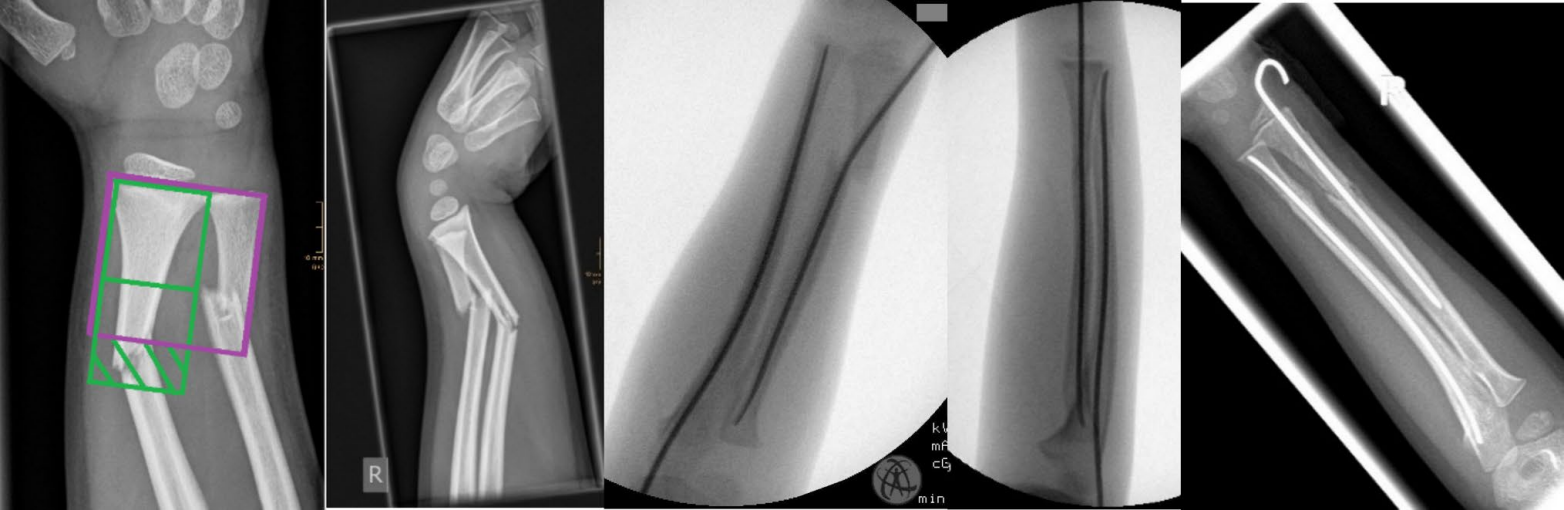
**a**–**e**. **a**, **b** a.p. and lateral radiograph of a diametaphyseal forearm fracture in a 9-year-old boy. The fracture lies within the diametaphyseal junction zone (DMJZ) as defined by a forearm fracture index between 1 and 2, but proximal to the metaphysis as defined by the AO-PCCF. This fracture can hence be categorized as DMRF+. **c**, **d** intraoperative a.p. and lateral radiographs after reduction and osteosynthesis with elastic stable intramedullary nailing (ESIN). The distal radial fragment presents with translation and ulnar deviation in the a.p. radiograph. **e** a.p. radiograph after removal of the ESIN osteosynthesis and reduction and transepiphyseal percutaneous intramedullary Kirschner-wire fixation (TEPIK) of the fracture. Angulation and translation of the distal radial fragment have disappeared

Figure 4 illustrates this phenomenon in a 9-year-old boy who presented with a diametaphyseal forearm fracture. The fracture was reduced and stabilized with the conventional ESIN technique. Due to insufficient reduction of the radial fragment with severe translation in the a.p. radiograph, the ESIN was removed and a TEPIK osteosynthesis was performed, leading to adequate reduction. Noteworthy, in the initial a.p. radiograph, no translation of the distal fragment was present, demonstrating how this fracture comprises the biomechanical characteristics typically seen in DMRF when ESIN osteosynthesis is performed.

**Figure 5:**
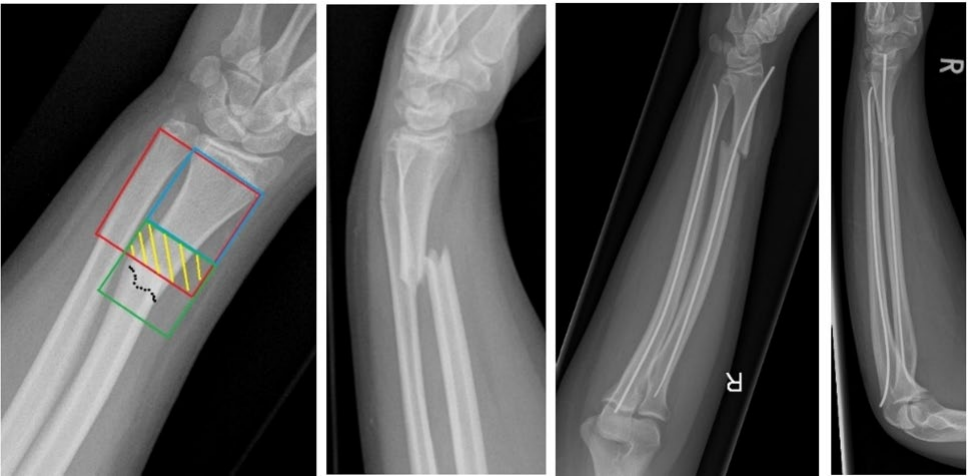
**a**–**d**. **a**, **b** a.p. and lateral radiograph of a diametaphyseal radius fracture in a 14-year-old girl. The black dotted line marks the fracture line. The red square marks the metaphysis, the yellow area marks the diametaphyseal junction zone (DMJZ) as defined by Lieber et al., the green square marks the DMJZ defined as the area of the radius with a forearm fracture index between 1 and 2. **c**, **d** postoperative a.p. and lateral radiographs after reduction and osteosynthesis with elastic stable intramedullary nailing (ESIN). The distal radius fragment presents with moderate translation and ulnar deviation in the a.p. radiograph

Figure 5 displays the case of a 14-year-old girl with a DMRF and ESIN osteosynthesis. Despite almost no translation or angulation in the initial a.p. radiograph, the postoperative a.p. radiograph shows the typical angulated ulnar deviation and radial translation of the distal radius fragment, illustrating the biomechanical behavior typically seen in DMRF. In this case, the dislocation was considered acceptable and no change of therapy was initiated. 1.5 years after the accident, rotation of the forearm was still reduced (supination/pronation: 40–0–90°) and the patient developed pseudarthrosis.

## Discussion

While the most widely used definition of the DMJZ is limited to the proximal part of the metaphysis as defined by the AO-PCCF, some authors have published different definitions of the DMJZ, such as those by Li et al. and Saliba et al. [2, 7, 9, 10, 12]. Both include a larger part of the radius and extend more proximally, as demonstrated in Fig. 1. Even though no argument has been given for why these larger definitions of a DMJZ were applied, they may reflect these clinicians’ experience that fractures slightly proximal to smaller definitions still exhibit biomechanical characteristics typical of DMRF.

Our results show that such fractures, which would have been considered diaphyseal according to the AO-PCCF classification but lie within the DMJZ as defined by an FFI between 1 and 2, that were treated with the conventional ESIN technique, did present with characteristic dislocation patterns postoperatively, typically seen in DMRF. While the fragments’ overall tilt was reduced, the number of fragments tilted towards the ulna increased from 3/7 to 5/7. Additionally, the distal radius fragment’s radial translation was increased after ESIN osteosynthesis, hence worsening the fracture’s position in this regard. This fracture behavior following conventional ESIN osteosynthesis of DMRF has been described numerous times before [2, 3]. It is important to note that the comparative analysis was performed in a very small cohort and is of exploratory nature. P-values are presented but must be interpreted with caution. The contextual interpretation of the results may support the authors’ hypothesis, but reliable inference of single test results is unlikely.

While its true clinical benefit remains unexplored, assessing the FFI in pediatric distal radius fractures might be one approach to help clinicians select a DMRF specific osteosynthesis technique in unstable DMRF [17]. Calculating the FFI to identify DMRF is simple; it includes the lateral radiograph, hence measuring an angulated fracture’s true distance to the growth plate, and radiographs of the wrist are sufficient as opposed to radiographs of the entire forearm, as needed for other definitions [8]. On top of that, the FFI can also be assessed via ultrasound [13]. With the FFI, a fracture’s location can be described accurately, which may make comparison of study results more valid.

Generally, classifications are considered clinically relevant when they have an impact on therapeutic management. While the ongoing discussion on the correct definition of the DMJZ may seem philosophical, it does influence clinical decision-making. The use of inappropriate osteosynthesis techniques in unrecognized DMRF with unsatisfactory reduction results may cause surgeons to address these fractures more aggressively. Recent studies have promoted plate osteosynthesis and external fixators as the most reliable osteosynthesis techniques for DMRF in older children [18, 19]. While both techniques provide good mechanical stability, in children minimally-invasive strategies should always be preferred if comparable results can be achieved. Volar plating leaves a considerable scar which can be avoided, and external fixators require good compliance and patients’ acceptance, which in childhood can be problematic. If a DMRF’s biomechanical behavior is understood, several less invasive techniques are available. Even in uncertainty with regard to the entity of the fracture (diaphyseal or diametaphyseal), a minimally invasive approach can be chosen, and an attempt at the conventional ESIN osteosynthesis can be made. If intraoperatively, after reduction and ESIN osteosynthesis, radial translation and ulnar deviation of the distal radius fragment are observed, the radial nail can be replaced by the double-bent nail described by Krohn [3]. Alternatively, the nail can be bent by 90° only once at the level of the entry point, hence eliminating elastic forces of the nail that would cause the typical radial translation and ulnar deviation of the distal radius fragment [7]. Surgeons should be aware of these techniques and avoid performing conventional ESIN osteosynthesis in DMRF.

Attention must be drawn to diametaphyseal greenstick fractures. Greenstick DMRFs that have not been completed may present with satisfactory reduction results upon conventional ESIN osteosynthesis. This may be due to the partly intact cortical bone, preventing the distal fragment’s radial shift and ulnar tilt. Nevertheless, it has been demonstrated that greenstick DMRF have a significantly increased risk of refracturing [17]. Completing greenstick fractures is mandatory when osteosynthesis is performed to lower the risk of refractures, compelling surgeons to then apply a DMRF specific osteosynthesis. Furthermore, the diametaphyseal location of the fracture may mislead surgeons into choosing the most distal entry point to the radius possible, inserting the nail through the Zone of Ranvier. This should be avoided at all times due to the danger of consequential growth disturbances [20–22]. One disadvantage of both modified ESIN techniques is that metal removal requires a second anesthesia.

Another osteosynthesis technique easily applicable is the TEPIK as published by Beck et al. [4]. Both the TEPIK and the two modified ESIN techniques present with excellent functional results, obey DMRF’s biomechanical requirements and are minimally-invasive, allowing good cosmetic results [3, 4, 7]. Concerns about cartilage damage or growth disturbances caused by TEPIK osteosynthesis have been refuted in a recent study [23]. Furthermore, treating distal radius fractures with an osteosynthesis method that addresses the biomechanical requirements of the DMJZ, even if the fracture does not behave like a DMRF, will not lead to insufficient osteosynthesis. Hence, one argument in favor of a larger definition of the DMJZ can be made in analogy to Pascal’s Wager. If we do not know whether a fracture needs a DMRF specific osteosynthesis or not, but we must decide on whether to perform a DMRF specific osteosynthesis or not, the following 4 options evolve: 1. The fracture does behave like a DMRF and we treat it like one. This will lead to good results. 2 The fracture does not behave like a DMRF, but we still treat it like one. This will also lead to good results. 3. The fracture does behave like a DMRF, but we do not treat it like one. This will lead to bad results. 4. The fracture does not behave like a DMRF, and we do not treat it like one. This will lead to good results. From a mathematical point of view, performing a DMRF specific osteosynthesis will lead to good results in 100% of the cases, while performing conventional ESIN osteosynthesis may lead to either good or bad results. In reality, a 100% success rate is of course unlikely as DMRF specific techniques carry complication risks just like all other osteosynthesis techniques do. Table 3 visualizes this “modified Pascal’s Wager”.

**Table 3:** Fourfold table visualizing 4 different scenarios in the treatment of diametaphyseal radius fractures (DMRF)

	DMRF+ do behave like real DMRF	DMRF+ do not behave like real DMRF
DMRF specific osteosynthesis	Stable osteosynthesis = good results	Stable osteosynthesis = good results
Conventional ESIN technique	Osteosynthesis fails = bad results	Stable osteosynthesis = good results

DMRF+, distal radius fracture with a forearm fracture index between 1 and 2 but categorized as diaphyseal according to AO-PCCF; ESIN, elastic stable intramedullary nailing

## Limitations

This study is limited by its retrospective nature and the small patient number. Potential bias cannot be excluded due to non-randomization regarding the choice of osteosynthesis. Routine follow-up did not include a standardized functional assessment. Even though biomechanical principles characteristic of DMRF could be demonstrated on radiographs of DMRF+ after conventional ESIN osteosynthesis, in practice, this led to a change in procedure in only one patient. Moreover, a comparison group of ‘true’ diaphyseal radius fractures (with an FFI > 2) is not provided.

## Conclusion

Defining the DMJZ through the FFI remains controversial, but the presented cases support the hypothesis that the DMJZ might extend a little further than more restrictive definitions suggest. Fractures located in this zone should be appreciated for their biomechanical behavior, namely ulnar deviation and radial translation of the distal radius fragment when treated with the conventional ESIN technique, designed for true diaphyseal forearm fractures [14–16]. Surgeons should be aware of this entity and be prepared to adapt their surgical technique intraoperatively to achieve the best possible result, while maintaining a minimally-invasive approach.

## Supplementary Information

Below is the link to the electronic supplementary material.

Supplementary file1 (DOCX 79 kb)

## Data Availability

All data presented is available from the corresponding author upon reasonable request.
