# Supplementary material for: Why it’s better to believe in a larger definition of the diametaphyseal junction zone in pediatric distal radius fractures
Source: Arch Orthop Trauma Surg. 2026 Jul 20;146(1):265. doi: 10.1007/s00402-026-06428-8 (PMC13385137; doi:10.1007/s00402-026-06428-8)
Supplement: Supplementary file 1 — Supplementary file1 (DOCX 79 kb) [file 402_2026_6428_MOESM1_ESM.docx]

**Supplementary Material**
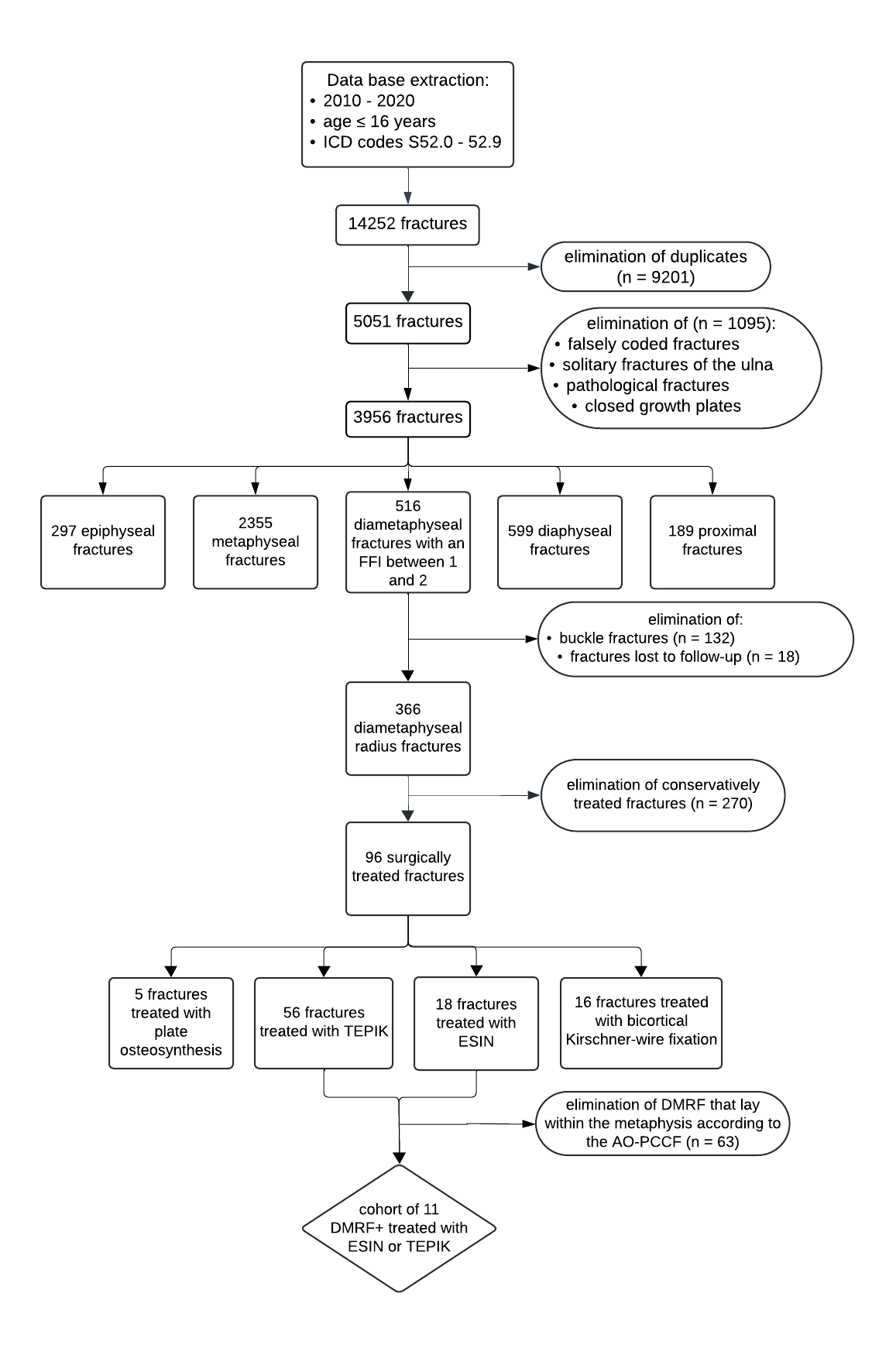


**Figure S1.** Flow diagram of patient acquisition. FFI, forearm fracture index; TEPIK, transepiphyseal percutaneous intramedullary Kirschner-wire; ESIN, elastic stable intramedullary nailing; DMRF, diametaphyseal radius fracture; DMRF+, diametaphyseal radius fracture outside the metaphysis; AO-PCCF, AO Pediatric Comprehensive Classification of Long Bone Fractures.
